# Properties and Limitations of eDNA Substrates for Terrestrial Animal Monitoring

**DOI:** 10.1111/1755-0998.70096

**Published:** 2026-01-23

**Authors:** Beilun Zhao, Tobias Andermann

**Affiliations:** ^1^ Mountain Ecological Restoration and Biodiversity Conservation Key Laboratory of Sichuan Province, Chengdu Institute of Biology Chinese Academy of Sciences Chengdu China; ^2^ Department of Organismal Biology Uppsala University Uppsala Sweden; ^3^ Science for Life Laboratory Uppsala University Uppsala Sweden

**Keywords:** conservation biology, DNA barcoding, ecological genetics, environmental DNA

## Abstract

Environmental DNA (eDNA) constitutes a valuable tool for monitoring terrestrial animal diversity, but outcomes are affected by multiple factors. Among these factors, the choice of sampling substrate and method is especially important and must be aligned with research objectives. We reviewed 245 published studies that utilise eDNA for terrestrial animal monitoring and compiled an overview of the most frequently used environmental substrates. Based on the reviewed literature, we provide a key description of each substrate, as well as its particular properties and limitations related to the detection of animal species across different spatial and temporal scales. We categorise these substrates into three groups: abiotic substrates (soil, water, air, sediment), biotic substrates (invertebrate samples, plant tissues, spiderwebs) and direct‐evidence substrates (scat, footprints, shelters, feeding sites). In addition, we identify several key challenges with the interpretation of eDNA‐based biodiversity monitoring, including false negatives and false positives, as well as the dynamics of spatial and temporal deviations. The latter concepts, which we propose and define in this review, describe the temporal and spatial discrepancies between the DNA source and its detection in a given sample. We reflect on how these temporal and spatial deviations are expected to affect eDNA data extracted from the different types of substrates and how knowledge of these dynamics can inform effective and accurate biomonitoring. In summary, this review provides a decision basis for designing terrestrial eDNA monitoring studies by summarising the properties and limitations of different substrates and contextualising the interpretation of results in light of substrate‐specific challenges.

## 1 Introduction

Terrestrial animals play critical roles in ecosystem functioning, driving essential processes such as nutrient cycling (McCary and Schmitz 2021), pollination (Ollerton et al. 2011), seed dispersal (Brockelman et al. 2022) and trophic regulation (McDermott 2025). As key components of food webs and ecosystem engineers, terrestrial animals maintain ecological structure, function and resilience (Blaum et al. 2011; DeVault et al. 2003). However, many species of terrestrial animals are currently facing severe threats from habitat loss, climate change and anthropogenic land‐use alterations (Munstermann et al. 2022; Outhwaite et al. 2022; Shin et al. 2022). In order to design effective conservation measures and evaluate the success of such efforts, it is essential to develop efficient, scalable and accurate ways of monitoring terrestrial animal diversity.

Traditional monitoring techniques such as field observation, camera trapping and live trapping have long been employed to monitor animals in their natural habitats (Karlsson et al. 2020; Lin et al. 2021; O'Connell et al. 2011). While these methods offer valuable insights into species presences, behaviours and abundances, they can be labour‐intensive, time‐consuming and logistically challenging, particularly for elusive or cryptic species (Burton et al. 2015; Leempoel et al. 2020). Other emerging technologies include automated acoustic and camera monitoring, satellite tracking and other remote sensing techniques, which complement traditional methods by providing additional data on animal presences (Huang et al. 2021; Lynch et al. 2013; Thomas et al. 2011). In addition to these methods, there is an increasing number of studies applying environmental DNA (eDNA) analysis for terrestrial animal monitoring. eDNA analysis involves detecting traces of genetic material shed by animals (and other organisms) into the environment, offering a non‐invasive, cost‐effective and highly sensitive approach to species detection and monitoring (Bohmann et al. 2014; Cowgill et al. 2024; Newton et al. 2025).

Despite its many advantages, eDNA‐based biodiversity monitoring faces several challenges (Bell et al. 2024; van der Heyde et al. 2022). Unlike other monitoring methods that rely on direct species sightings, eDNA constitutes an indirect detection method, usually without directly observing the specimen that deposited the DNA. Moreover, DNA can move through the environment and can persist for long times, complicating the interpretation of eDNA sampled at a given place and time. A useful way to conceptualise these challenges is through the framework of the ecology of eDNA, which emphasises four key processes: the origin of genetic material shed by organisms (e.g., faeces, skin cells, or bodily fluids), its state once in the environment (ranging from intact cells to free DNA fragments), its transport through different substrates such as soil, water or air, and its eventual fate as it degrades under biotic and abiotic influences (Barnes and Turner 2015). This highlights that eDNA is not a static signal, but a dynamic biological entity shaped by ecological interactions that determine when, where and how DNA is detectable. These ecological interactions differ substantially between substrates, influencing both the detectability of organisms and the spatial and temporal interpretation of their detected presence in eDNA samples (Bell et al. 2024; van der Heyde et al. 2022).

This review presents an application‐oriented overview of the current state of knowledge about eDNA substrates commonly used for terrestrial animal monitoring, by summarising the information from 245 selected studies. We begin with a systematic overview of the challenges of false positives and false negatives, and introduce the concepts of spatial and temporal deviation. We then introduce different eDNA substrates in the context of their utility for terrestrial animal monitoring, emphasising their ecological properties and limitations. Rather than seeking a universally ‘most effective’ substrate, we emphasise that substrate choice is dependent on the ecological context and taxonomic focus of a given study. Through summarising the current knowledge and results from specific case studies, this review provides an overview of the methodological biases that may shape the outcomes of eDNA monitoring efforts and the trade‐offs that practitioners should consider when selecting a given substrate or combination of substrates. Aside from informing future study design, the information provided in this review helps interpret the results of eDNA‐based terrestrial animal monitoring datasets.

## 2 Literature Review

### 2.1 Methods

Our literature search strategy was designed to capture relevant studies on terrestrial animal monitoring that incorporated eDNA. We define eDNA as genetic material collected from environmental substrates or surfaces, including cases where the target species is detected through other species, such as using invertebrates (e.g., mosquitoes) to detect mammals. Studies that required lethal or invasive sampling of vertebrates, for example, studies that analyse mammalian stomach contents for reconstructing dietary components, were excluded, as these methods are generally difficult and morally questionable to apply at scale. We also excluded laboratory‐only studies such as controlled experiments on eDNA properties or methodological comparisons which are not directly linked to field monitoring. Bulk samples such as Malaise trap samples for insects and studies focused on ancient DNA were also excluded. This systematic review followed the PRISMA (Preferred Reporting Items for Systematic Reviews and Meta‐Analyses) guidelines to ensure comprehensive and unbiased literature coverage.

We conducted a systematic search in Scopus (www.scopus.com) on August 30, 2025, using the following Boolean search string: (eDNA OR ‘environmental DNA’ OR ‘invertebrate DNA’ OR iDNA OR ‘air DNA’ OR ‘airborne DNA’ OR ‘airborne environmental DNA’ OR ‘DNA trace*’) AND (animal OR invertebrate* OR vertebrate* OR bird* OR mammal* OR reptile* OR arthropod* OR insect* OR bat* OR species) AND NOT (mussel OR bacteria* OR fungi* OR marine* OR ‘ballast water’ OR fish* OR ‘coral reef*’ OR amphibian OR crab* OR ‘coral reef*’ OR plankton OR benthic* OR biofilm* OR virus* OR disease* OR infection* OR antibiotic) AND NOT (‘laboratory setting*’ OR aquarium* OR mock* OR pipeline* OR medical) AND NOT (symposium OR conference* OR bibliography* OR review*). This search returned 963 articles. We refined the collection by only keeping articles that reported the use of eDNA for terrestrial animal monitoring, based on the information in the abstract, leaving a remaining 191 articles. To ensure comprehensive coverage, we supplemented this search through consultation with domain‐experts (a total of seven reviewers) for potentially missed studies, which were manually added to the literature list. This led to a total of 245 publications that were included in this review (Table S1).

### 2.2 Summary

**FIGURE 1:**
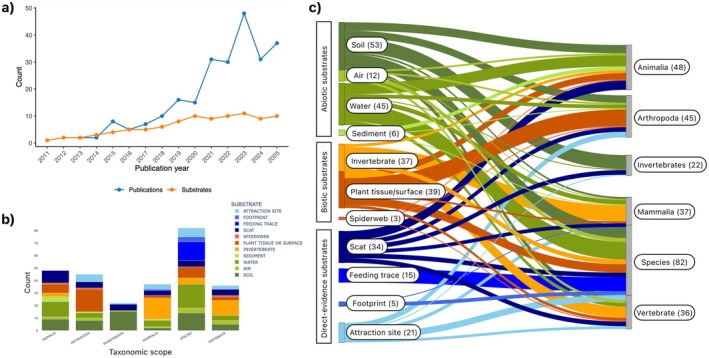
Overview of publication trends applying environmental DNA (eDNA) for terrestrial animal monitoring. (a) Annual number of publications employing eDNA (blue) and the number of different eDNA substrates used (orange), from 2011 to August 30, 2025. (b) Number of publications across different eDNA substrates per taxonomic target group. The target group refers to the taxonomic range that was targeted in the study, leading to sometimes overlapping or encompassing categories (e.g., ‘Vertebrates’ and ‘Mammalia’). Several studies were focused on specific taxonomic subsets of mammals or arthropods, which, for simplicity, were rolled up to the broader taxonomic groups ‘Mammalia’ and ‘Arthropoda’ respectively, except if the target of a study was an individual species, which we represent as its own category (‘Species’). (c) Sankey diagram visualising the taxonomic scope targeted by each eDNA substrate across the reviewed articles.

**FIGURE 2:**
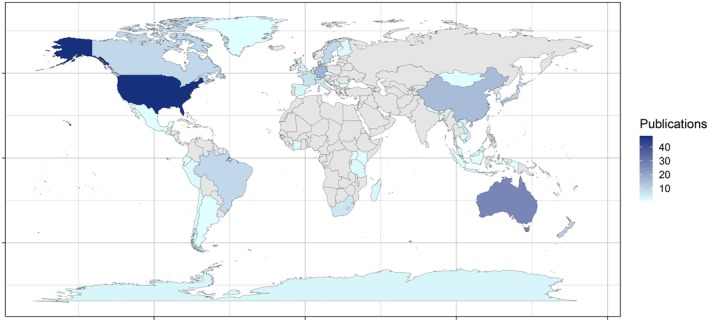
Heatmap showing the number of publications by country where the fieldwork was carried out. Six publications were excluded from this figure due to non‐specific (e.g., ‘Europe’ or ‘Africa’) or missing location data in the main text.

The collection of selected articles spans from 2011–2025 and involves a total of 13 different substrate types from which eDNA has been extracted (Figure 1). The taxonomic scope of these studies ranges from broad groups (all animals) to more specific taxonomic subsets, including vertebrates, invertebrates, arthropods, mammals, all the way to individual species (Figure 1b). Taxonomic ranks below class (namely order, family and genus) were merged at class level, except when the study focused on the detection of individual species, which we kept as its own category of taxonomic scope, namely ‘species’. The studies encompass multiple countries (Figure 2), with the United States representing the most selected studies (*n* = 48), followed by Australia (*n* = 27). Ecosystems that were targeted in these eDNA studies include natural environments such as forests, coastal habitats, grasslands and mountain ecosystems, as well as artificial ecosystems, such as urban areas, zoos, farmlands and gardens. While most studies involved one‐time sampling, some conducted repeated surveys across multiple seasons or years.

## 3 Challenges

**FIGURE 3:**
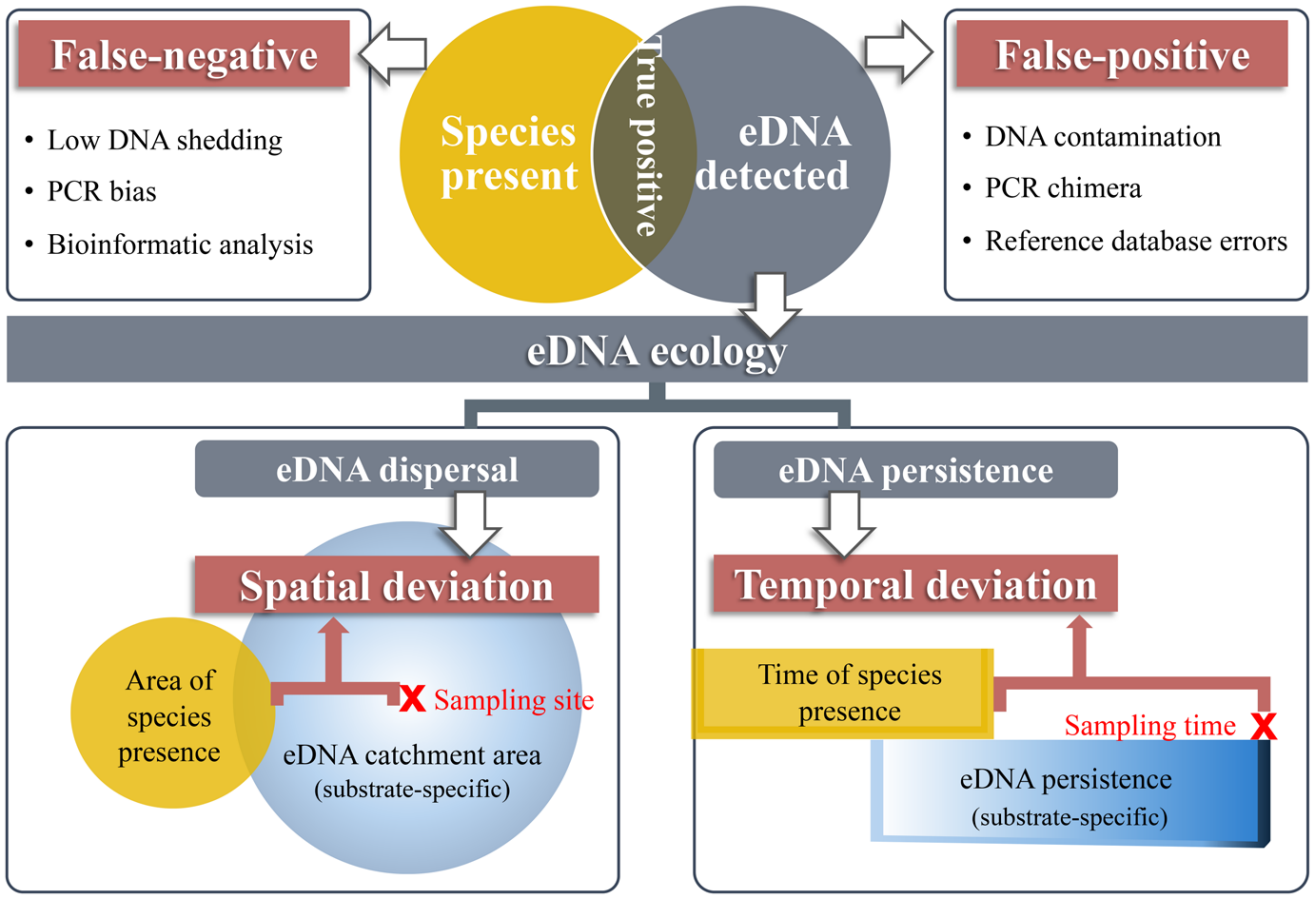
Challenges of environmental DNA (eDNA)‐based biomonitoring. These challenges include detection of false negatives, false positives and deviations between DNA source and site of detection. Spatial deviation (bottom left) arises from eDNA dispersal, where DNA of species is detected at a distance from where it was deposited by the source organism. Temporal deviation (bottom right) occurs when eDNA persists over time, leading to the detection of DNA that has been deposited at some time before the sampling event.

There are several key challenges inherent to eDNA‐based biodiversity monitoring, which are repeatedly observed in the reviewed literature. These include the possibility of false positives (species detected that are not actually present) and false negatives (species present but not detected), which can arise from multiple factors at different stages of the workflow. Another important challenge is the possible travel of eDNA in the environment and the persistence of DNA through time, leading to a discrepancy between the actual location or time of an organism's presence (the DNA source) and the location or time at which its DNA is detected in an environmental sample (Figure 3). Although earlier work on eDNA ecology (Barnes and Turner 2015) has conceptualised the movement of DNA through the environment, this expectation is rarely sufficiently emphasised or acknowledged in eDNA‐based monitoring studies. To address and draw attention to this gap, we introduce the terms ‘spatial deviation’ and ‘temporal deviation’ to describe these common processes, which are likely to affect most eDNA‐derived biodiversity data. Below, we define these challenges and summarise current knowledge about their prevalence and possible extent, as well as possible mitigation strategies.

### 3.1 False Negatives

False‐negative results occur when a target species is present at the sampling site but is not detected by the eDNA analysis of samples collected from that site (Guillera‐Arroita et al. 2017). This can result from low eDNA shedding rates, substrate choice and environmental conditions. For instance, Johnson, Barnes, et al. (2023) and Johnson, Katz, et al. (2023) demonstrated that in comparison to camera traps, eDNA was better at identifying Diptera and Thysanoptera flower‐visiting pollinators but worse for Hymenoptera and Lepidoptera, missing up to one fourth of the species detected with the camera traps. This suggests that some arthropods may release too little DNA to be detected, likely due to their hard exoskeleton. Similarly, eDNA detection of very low‐density species such as pythons (Hunter, Meigs‐Friend, et al. 2019) results in inconsistent and unreliable detection, particularly when using non‐targeted sampling, such as general soil sampling in a target area (Duarte et al. 2023). False negatives may also arise during the eDNA sample processing steps, for example when selected primers fail to amplify certain target species or when low‐abundance DNA is not sufficiently amplified during PCR.

Sampling substrates that are frequently contacted by the target species increases detection rates. For example, Katz et al. (2021) found that soil inside artificial shelters led to the detection of a threatened snake in 100% of cases, compared with only 45% detection success for general soil samples from surrounding sites. Similarly, sediments from log hollows, which animals frequently visit, provide higher species recovery than general soil samples (Newton et al. 2022; Ryan et al. 2022).

Environmental conditions further influence false‐negative results. High temperatures or prolonged sunlight exposure accelerate eDNA degradation, reducing detection success (Kucherenko et al. 2018). Rainfall and wind can also affect eDNA detectability. For instance, even light rain can wash away eDNA from plant surfaces, resulting in the disappearance of target DNA and therefore a lack of detectability of a target species on this surface (Valentin et al. 2021). The impact of environmental factors such as sun exposure varies vastly among substrates, which we cover in more detail below.

### 3.2 False Positives

We define false positives as the inference of a species detection that is incorrect, that is, the DNA of the species in question is not actually present in the raw sample (sensu Darling et al. 2020). False positives can arise from contamination during field collection or laboratory processing, or from incorrect DNA sequence annotation due to limitations or errors in reference databases. Rigorous laboratory protocols, inclusion of negative controls and careful bioinformatic annotation using high‐quality, curated reference databases are therefore critical to minimise such errors. False positives may also result from barcode‐sharing, where multiple species share identical sequences in the targeted DNA region, leading to the potential of picking the incorrect sequence match for annotation. Using longer barcode regions in metabarcoding studies can help mitigate this issue. In addition, PCR‐generated chimeras are considered false positives, as they produce sequences not biologically present at the sampling site; although these usually don't lead to a false detection of a specific species, these artifically generated sequences can inflate the estimated number of species proxies within a given group. The detection of DNA fragments originating from a different location or time relative to the sampling event is categorised under ‘spatial and temporal deviations’ below. We do not consider those cases as false positives, as the DNA molecules of the species in question are in fact present in the sample and can sometimes represent an intended target of the monitoring effort.

### 3.3 Spatial Deviation

Spatial deviation occurs when eDNA is transported from its original source to the sampling site through various environmental processes such as wind, rain, flowing water, or animal movement, complicating accurate localised assessments of species communities (Foucher et al. 2020). For instance, airborne eDNA largely reflects local species, but can also detect genetic material from spatially distant species transported via pollen or spores (Lynggaard et al. 2024; Sullivan et al. 2023). Similarly, water flow can carry eDNA hundreds of meters to several kilometres downstream, depending on waterbody characteristics such as flow velocity and benthic substrate, leading to detections far from the source (Fremier et al. 2019; Shogren et al. 2018). Moreover, eDNA can be dispersed by rainfall, for example, from the forest canopy to the surface soil, potentially resulting in spatial deviation (Macher et al. 2023). Further, animal activities can contribute to eDNA transfer, leading to spatial deviations. For example, fish eDNA can be carried to terrestrial environments via the scat of terrestrial animals that consume fish, such as many shore‐birds (Sullivan et al. 2023). Considering these dynamics is important for the interpretation of biodiversity data derived from eDNA, as not all species detected can be assumed to be present at the sampling site. Rather the detected species pool is likely a combination of locally present species and DNA material from other species that have been transported to the sampling site via a combination of the described mechanisms. As we outline below, some substrates are generally more strongly affected than others by this mechanism of spatial deviation.

### 3.4 Temporal Deviation

Temporal deviation occurs when eDNA persists after deposition, leading to detections long after a species was present at a site. Depending on the substrate and environment, eDNA can remain detectable from several hours to thousands of years (Pedersen et al. 2015; Wang, Pedersen, et al. 2021; Wang, Zhao, et al. 2021). This DNA persistence varies across substrates and is influenced by environmental factors like temperature, UV radiation, pH, salinity and microbial activity (Jo and Minamoto 2021; Joseph et al. 2022; Zhao et al. 2023). Current evidence suggests eDNA persists longer in solid abiotic substrates, followed by biotic substrates, and shortest in water, with DNA degradation being fastest under wet and warm conditions. Therefore, the likely timeframe of DNA deposition can often be inferred based on the type of eDNA substrate analysed. For instance, soil eDNA can reveal species that were present more than 100 days earlier (Leempoel et al. 2020), and mammalian DNA has been detected in leeches over 4 months after ingestion of host blood (Schnell et al. 2012). In contrast, eDNA in water typically persists only several days to weeks under above‐freezing conditions (Barnes et al. 2014; Joseph et al. 2022; McCartin et al. 2022). Temporal deviation can in some cases be a desirable feature, as it allows, for example, to capture within a single sampling event a variety of species that were present at different times at a site (‘substrate memory’, sensu Foucher et al. 2020).

## 4 eDNA Substrates and Their Properties and Limitations

Over time, an increasing variety of eDNA substrates have been utilised in terrestrial animal monitoring, each with distinct advantages and limitations (Bell et al. 2024; van der Heyde et al. 2022). Substrate choice is crucial because it determines which species can be detected and influences the degree of expected spatial and temporal deviation, as well as the potential of false positives and false negatives (Newton et al. 2022; van der Heyde et al. 2020; Wheat et al. 2016). Thus, understanding the specific properties and limitations of different eDNA substrates is essential when planning effective field sampling for biodiversity monitoring.

We categorise eDNA substrates into three groups (Table 1 and Figure 1c): Group A comprises abiotic substrates (soil, water, sediment and air) which accumulate eDNA through passive environmental deposition. Group B refers to biotic substrates, including organismal samples, such as invertebrates (e.g., flies or mosquitos), plant tissues (e.g., flowers or leaves) and spiderwebs, where eDNA is deposited through direct or indirect ecological interactions with living organisms (e.g., feeding, pollination, movement, entrapment). Group C consists of direct‐evidence substrates, referring to either physical evidence of species presence, such as scats, footprints and feeding traces, or to artificial and natural attraction sites, such as shelters, nests, water tanks, salt‐licks, or bait stations. The distinction between these categories is not always straightforward, but reflects our attempt to group the different types of eDNA substrates thematically and by their inherent properties. Note that this categorisation is limited to currently applied eDNA substrates reported in the literature; we anticipate that additional substrates will be employed for future biodiversity monitoring.

**TABLE 1:** Overview of eDNA substrates commonly used for terrestrial animal monitoring. The provided reference gives an example of each substrate.

Substrate	Brief description	Number of publications	References
**Group A: Abiotic substrates**	Abiotic media that accumulate eDNA through passive environmental deposition without requiring biological intermediaries		
Soil	Direct collecting surface soil	53	Lynggaard et al. (2022), Tetzlaff et al. (2024)
Water	Filter water in the field	45	Lin et al. (2021)
Air	Set air filtration facilities and collect filters after a period	12	Lynggaard et al. (2022)
Sediment	Direct collecting surface sediment	6	Palacios Mejia et al. (2021)
**Group B: Biotic substrates**	eDNA is acquired through ecological interactions between living organisms		
Invertebrate	Search and collect invertebrates, such as leeches or mosquitoes, with the goal to detect species that these invertebrates commonly interact with	37	Lynggaard et al. (2019)
Plant tissue or surface	Collect plant tissues such as flowers, or swabs of plant structures such as tree bark or leaf surface	39	Johnson, Katz, et al. (2023), Allen et al. (2023)
Spiderweb	Use sterile swabs to collect spiderweb	3	Newton et al. (2024)
**Group C: Direct‐evidence substrates**	Direct physical evidence or attraction sites of specific target species		
Scat	Collection or subsampling (e.g., through swab) of the scat	34	Levesque‐Beaudin et al. (2023)
Attraction site	Natural or artificial structures that attract animals, such as shelters, nests, or feeding sites	21	Kim et al. (2022), Newton et al. (2022)
Feeding trace	Plant or animal tissue with animals' feeding traces such as bite marks	15	Monge et al. (2020)
Footprint	Direct sampling of the footprints of animals, for example, in snow or mud	5	De Barba et al. (2024)

### 4.1 Abiotic Substrates

#### 4.1.1 Soil

Soil samples are commonly used as a source of eDNA for monitoring various taxa in terrestrial ecosystems (Bienert et al. 2012; Kirse et al. 2021; Ritter et al. 2018), applied in 53 of the 245 collected studies (Table 1). Animals shed genetic materials, such as skin cells and hair, which can accumulate on surrounding surfaces (e.g., tree bark) and subsequently transfer to soil through natural processes such as wind and rainfall (Lunghi et al. 2022). This accumulation of genetic materials in soil provides valuable information on species presence (Marquina et al. 2019; Ritter et al. 2018). Soil samples can be collected using methods such as coring, surface sampling, or vacuuming, often in areas of high species activity or habitats where target species are likely to deposit genetic material (Allen et al. 2023; Lunghi et al. 2022; Ryan et al. 2022). Soil tends to retain DNA locally, leading to higher turnover rates between nearby soil samples compared with other abiotic substrates, such as air or water. This introduces some stochasticity in being able to detect a specific target species, often requiring multiple soil samples to representatively inventory a given site.

##### 4.1.1.1 Properties and Limitations

Particularly due to the high local structuring and turnover of soil eDNA, a common issue is that of false negatives. Several studies have reported cases where species detected by conventional methods, such as camera traps, were missed by soil eDNA analysis (Leempoel et al. 2020; Lin et al. 2021; Yang et al. 2014). In addition, false negatives may also result from PCR inhibitors common in soil, such as humic acids, which reduce amplification efficiency and introduce detection biases (Arbeli and Fuentes 2007). Another defining property of soil is its generally long DNA persistence potential, leading to temporal deviations (Figure 3). eDNA can persist in soil for several months to years (Leempoel et al. 2020) because DNA molecules bind to soil particles, which protects them from degradation by environmental DNases (Cai et al. 2006; Pietramellara et al. 2009). Under the right conditions, such as cold and dry environments not exposed to direct sunlight, this can lead to the preservation of eDNA over thousands of years (ancient eDNA), allowing the detection of even extinct species such as mammoths in eDNA derived from soil samples (Wang, Pedersen, et al. 2021; Wang, Zhao, et al. 2021).

#### 4.1.2 Water

Water eDNA is the second most commonly employed substrate for monitoring terrestrial animals (45/245 studies, Table 1). Terrestrial animals directly interact with water bodies through activities such as drinking, swimming, laying eggs, or developing larval stages, which introduces their DNA into the aquatic eDNA pool (Gutiérrez‐López et al. 2023). In addition, DNA from terrestrial species can end up in aquatic environments via rain, streams or wind (Macher et al. 2023; Mena et al. 2021; Villacorta‐Rath et al. 2023). Results from aquatic eDNA are generally more reproducible than soil eDNA, as water is a more homogeneous medium, allowing genetic material to disperse more evenly (Mauvisseau et al. 2022; Taberlet et al. 2018; Thomsen and Willerslev 2015). As a result, fewer replicates from the same site are needed for inventories, reducing workload and the risk of false negatives (Sakata et al. 2021). Water samples are typically collected via filtration, which requires specialised equipment (Goldberg et al. 2016; Mächler et al. 2018; Takasaki et al. 2021). Filters usually have pore sizes larger than 0.2 μm, which may fail to capture highly degraded DNA bound to small particles, as these pass through the filter undetected, limiting the detection to recently shed genetic material of longer fragment length (Barnes et al. 2014; Zhao et al. 2021).

##### 4.1.2.1 Properties and Limitations

Water can contain suspended particles such as soil and organic matter, which can complicate detection because DNA bound to particles will persist longer than free‐floating DNA in the water column, leading to differences in temporal deviation (Egeter et al. 2018). Flow rates in streams, rivers and lakes, along with local climate conditions, can significantly influence the spatial and temporal catchment area of species in water eDNA (Eichmiller et al. 2016; Shogren et al. 2017, 2018). Therefore, compared with soil, water eDNA generally provides broader species coverage, with lower temporal but higher spatial deviations.

#### 4.1.3 Air

Air (12/245 studies, Table 1) serves as an effective medium for biomonitoring through eDNA sampling, with airborne eDNA originating from various sources including shed skin cells, hair, feathers and specifically adapted dispersal structures such as pollen and spores (Johnson, Fokar, et al. 2021). Airborne eDNA collection methods typically employ either passive or active filter‐based samplers, with adjustable parameters such as filter pore size and collection duration. Active samplers, such as high‐volume air pumps, can also be configured to adjust air‐throughput (Garrett, Watkins, Simmons, et al. 2023; Johnson and Barnes 2024; Lynggaard et al. 2024). Air sampling generally requires more labour and time, compared to soil and water sampling, due to equipment setup and filtration duration. Existing and permanently installed air monitoring stations can significantly reduce the effort involved in air eDNA collection. In various places, air filtration stations have been installed over the decades for other reasons than eDNA sampling, which can sometimes constitute an unexpected data source for the retroactive monitoring of past biodiversity with the potential for creating long‐term biodiversity time series data (Littlefair et al. 2023; Lynggaard et al. 2024; Sullivan et al. 2023). For example, a study in Sweden demonstrated that air filters employed for aerosol monitoring and stored in airtight containers can be readily used to extract eDNA and trace biodiversity over the past three decades (Sullivan et al. 2023).

##### 4.1.3.1 Properties and Limitations

Airborne eDNA can travel long distances, influenced by wind and atmospheric conditions. A general pattern is that filters placed up high above the ground capture eDNA over a larger spatial range, compared with sampling closer to the ground (Johnson and Barnes 2024). This allows to an extend for sampling design to control for the spatial range of the expected catchment area. One study in the UK demonstrated that air eDNA could effectively capture biodiversity patterns across a continental scale, illustrating its potential for large‐scale ecological monitoring (Littlefair et al. 2023). The wide spatial range that can potentially be captured by airborne eDNA allows for the effective detection of invasive or endangered species with low population densities, which might be overlooked by other monitoring methods (Johnson, Cox, et al. 2021; Johnson, Fokar, et al. 2021). Future manipulative or experimental studies, such as systematically sampling at multiple distances and directions from target organisms, would help us better understand airborne eDNA transport and its ecological implications.

#### 4.1.4 Sediment

Sediment eDNA is only in rare cases applied for terrestrial animal monitoring (6/245 studies, Table 1). In this context, we refer to sediment as the solid substrate that is covered by a water body. DNA of terrestrial animals can end up in the sediment through the same interaction mechanisms as described above for water. Some of the DNA fragments in the water column eventually settle in the sediment, where they can persist for a longer time, as they bind to sediment particles that protect the DNA from microbial digestion and mechanical forces present in the water column (Holman et al. 2019; Palacios Mejia et al. 2021; Siano et al. 2021). This property can lead to the preservation of historical eDNA, particularly in deep layers, enabling the reconstruction of past species communities from sediment eDNA (Parducci et al. 2017; Willerslev et al. 2003). For example, Siano et al. revealed shifts in coastal biodiversity in response to agricultural pollutants released during *World War II* by analysing deep sediment cores (3–12‐m depth, sampled 31–334‐cm cores) (Siano et al. 2021). This capability makes sediment eDNA a promising tool for retrospective biodiversity analysis, though further methodological refinements, particularly to improve temporal resolution, are still needed.

##### 4.1.4.1 Properties and Limitations

The longer temporal deviation of sediment eDNA compared with water makes sediment eDNA more suitable for the reconstruction of past species communities, but at the same time limits its applicability for monitoring of species that are currently active in the environment. Further, spatial deviation is expected to play a significant role for this substrate, particularly in case of sediments in water bodies with a current, as DNA can be transported over long distances and settle in the sediment kilometres away from where it was disposed. However, at present, there is little information available in the existing literature to properly evaluate the possible extent of spatial deviation for this substrate.

### 4.2 Biotic Substrates

Although abiotic eDNA substrates are generally straightforward to locate and collect (barring potential site‐specific complications) and provide broad taxonomic coverage, they may fail to detect species presence in specific locations or during particular time periods, especially for elusive taxa or within complex ecosystems (Amavet et al. 2023; Ruiz‐Ramos et al. 2023). To overcome this potential limitation, targeted eDNA sampling methods have been developed that use biotic substrates such as invertebrates or plant material, which are more likely to have direct contact with the focal species. These substrates acquire DNA through interspecific interactions such as blood‐feeding and pollination and can therefore be utilised to detect the presence of a given focal species, without having to directly encounter that species (Evans and Kitson 2020; Harper et al. 2023; Johnson, Katz, et al. 2023).

#### 4.2.1 Invertebrates

The use of invertebrate eDNA (iDNA) for monitoring a variety of other associated taxa has gained popularity in recent years (Carvalho et al. 2022; Ji et al. 2022; Kocher et al. 2017) (37/245 studies, Table 1). iDNA has been obtained from diverse taxa, including blood‐feeding parasites like leeches, mosquitoes, biting flies and ticks, as well as dung‐feeding insects such as flies and dung beetles (Bohmann et al. 2014; Ji et al. 2022). While feeding, these invertebrates ingest genetic material from their vertebrate hosts, which can persist in their gut contents and remain detectable for several months (Cutajar and Pulsford 2023; Schubert et al. 2015). This method is effective for detecting a wide variety of vertebrate species (Cutajar and Pulsford 2023; Kocher et al. 2017; Lynggaard et al. 2019). Due to the generally limited movement range of invertebrates, iDNA typically detects species that were present within a few hundred meters of the sampled invertebrates, reflecting a more precise spatial representation of species presence compared with water or air samples (Saranholi et al. 2023). However, iDNA sampling is labour‐intensive, often requiring the collection of hundreds to thousands of invertebrate specimens to increase the chance of detecting the target species (Cutajar and Pulsford 2023; Fernandes et al. 2023; Massey et al. 2022; Saranholi et al. 2023). An important factor to consider is the selection of invertebrate species for sampling. Although different invertebrate species may have similar spatial detection ranges (Saranholi et al. 2023), the diversity of vertebrate species identified can vary significantly depending on the invertebrate's ecological niche and host specificity. For example, carrion flies often reveal more vertebrate species than sandflies or mosquitoes do (Massey et al. 2022).

##### 4.2.1.1 Properties and Limitations

Detecting a given (vertebrate) species through sampling eDNA derived from invertebrates requires these invertebrates in some form to interact with said species. This will enable the detection of some target species but might lead to missing others, even if they are present at the site. As a result, iDNA‐based inventories have the potential to differ substantially from the vertebrate species observed by visual inventory methods (Abrams et al. 2019; Massey et al. 2022). In cases where such ecological interactions are known and where the goal of the study is the monitoring of a specific set of target species that are known to be in interaction with a given set of invertebrate species, this method can provide reliable detection of these target species. An additional consideration is that the eDNA ecology, including the potential spatial and temporal deviation, can vary vastly among the various sources of invertebrate‐derived eDNA, as these mechanisms are affected by the ecology and behaviour of the given invertebrate species that is being sampled. Spatial deviation of eDNA is directly affected by the movement range of the invertebrate species. Regarding temporal deviation, invertebrate‐derived eDNA (iDNA) is likely to degrade faster on the sun‐exposed proboscis of a flying insect compared with the iDNA derived from a leach whose behaviour keeps it largely hidden from direct sun‐exposure. This is a speculative example, as these dynamics are currently still poorly understood, requiring more targeted research.

#### 4.2.2 Plant Tissues and Surfaces

DNA collected from plant surfaces, whether through direct sampling of plant tissues or through surface swabbing, provides a valuable and frequently applied method of monitoring animal species that interact with plants (39/245 studies, Table 1). For instance, pollinators visiting flowers often leave traces of their DNA on floral tissues (Evans and Kitson 2020), which can be detected through eDNA sampling of the floral tissue (Jønsson et al. 2023; Kestel et al. 2023; Newton et al. 2023). This approach has successfully detected a wide range of organisms, primarily arthropods (Figure 1b) (Johnson, Katz, et al. 2023; Jønsson et al. 2023; Thomsen and Sigsgaard 2019), as well as birds and mammals (Jønsson et al. 2023; Newton et al. 2023). A related method, plant surface swabbing, involves rolling or wiping plant surfaces, such as bark or leaf surfaces, with a paint roller or similar tool to collect tissue fragments or other genetic trace material (Lynggaard et al. 2023). This technique has been successfully applied in forests and gardens to detect a diverse range of animal taxa (Aucone et al. 2023; Harper et al. 2023; Lyman et al. 2022). For example, bark swab eDNA detected 15 of 25 previously recorded arboreal mammal species, while soil eDNA at the same sites detected only eight (Allen et al. 2023).

##### 4.2.2.1 Properties and Limitations

Plant surface eDNA can provide a powerful approach to detect specific animal species communities, in some cases providing more effective monitoring of terrestrial animals than other eDNA substrates such as soil (Allen et al. 2023), as well as outperforming alternative inventory methods such as camera traps (Johnson, Katz, et al. 2023). However, this eDNA substrate is mainly of high utility for specific groups of animals, depending on their ecology and behaviour. Groups such as pollinators and arboreal species can be efficiently monitored through this method, while other groups with limited direct plant interactions go largely undetected. Further, this substrate is likely to lead to false negatives, even for the target groups of pollinators and arboreal species, as environmental factors such as rain can wash away deposited DNA (Johnson, Katz, et al. 2023).

#### 4.2.3 Spiderwebs

Spiderweb eDNA sampling (3/245 studies, Table 1) takes advantage of spiderwebs' natural capacity to trap DNA from organisms that either brush against or become entangled in the web (Corse et al. 2019; Gregorič et al. 2022; Xu et al. 2015). Due to their widespread distribution and targeted placement to efficiently capture other arthropods, spiderwebs serve as excellent and easily accessible substrates for assessing arthropod diversity across ecosystems (Gregorič et al. 2022). The adhesive nature of spider webs also allows them to act as natural air filters, capturing genetic material from a wide range of taxa, originating from scales, hairs, exoskeleton fragments and bodily fluids. As a result, spiderweb eDNA offers valuable insights into the biodiversity of an area, detecting not only arthropods but also other animal phyla, including vertebrates (Newton et al. 2024).

##### 4.2.3.1 Properties and Limitations

Spiderwebs constitute a high‐utility eDNA substrate, as they are usually easy to locate and to sample, and effectively capture and bind a large variety of biological tissues and DNA. Given their natural ability to bind even the smallest particles, they are likely to pick up DNA carried over longer distances through wind, making them prone to spatial deviation. Their often temporary and fragile nature might suggest low potential for temporal deviation, yet spiders are known to recycle damaged parts of their webs (Opell 2021), which might allow DNA to persist through multiple rebuilds of the web. However, more research is needed to test how long after an organism's presence DNA can still be detected in a given spiderweb.

### 4.3 Direct‐Evidence Substrates

Substrates containing direct biological traces of target species (direct‐evidence substrates) may improve detection rates and reduce false negatives in studies that target one or a small number of species. These substrates can be classified into two main types based on their association with the target species: (1) direct physical traces of specific species, including scats, feeding traces and footprints; as well as (2) attraction sites where species return repeatedly, including bird nests and tree hollows, or purpose‐built setups such as drinking‐water tanks, salt‐licks, or bait stations.

#### 4.3.1 Physical Traces (Scats, Feeding Traces and Footprints)

Materials with physical traces left by animals have been effectively used as eDNA substrates, enabling species detection with high specificity. Scats have proven to be a particularly valuable eDNA source (34/245 studies, Table 1), as they offer genetic evidence of the depositing animal, as well as about its diet. This provides valuable insights into species presence, population dynamics, food networks and predator–prey interactions (Nørgaard et al. 2021; Pérez‐Mellado et al. 2011). Scats typically remain visible and thus sampleable for days to months, with the duration depending on environmental conditions such as temperature and humidity, which impact microbial decomposition (Quasim et al. 2018; van der Heyde et al. 2021). Other examples of physical trace substrates include footprint eDNA sampling from snow tracks for mammalian species identification (5/245 studies) (Kinoshita et al. 2019), as well as feeding traces (15/245 studies) such as leaf mines for monitoring plant pests (Pirtle et al. 2021) or residual saliva on partially consumed Pacific salmon for tracking and identifying brown bears (Wheat et al. 2016).

##### 4.3.1.1 Properties and Limitations

Sampling direct physical traces of target species can often be easier than observing the actual species, particularly in case of rare or elusive species, but often still requires substantial field effort to locate and sample eDNA from these traces. While this method generally provides high detection accuracy, it is usually limited to a single or small set of target species that can be surveyed by this method. As most of these physical traces are located at the site of species interactions and don't persist for a very long time, this method is generally unlikely to be affected by substantial spatial or temporal deviation; therefore, providing a more precise species detection in space and time compared with other substrates such as soil.

#### 4.3.2 Attraction Sites (Natural or Artificials Shelters and Feeding Sites)

To mitigate the difficulties of locating evidence of moving species, natural or artificial shelters and feeding sites provide a more practical alternative for eDNA collection, as they constitute sites that species repeatedly return to (attraction sites, 21/245 studies, Table 1). For instance, log hollows have proven effective for vertebrate monitoring, often leading to better detection efficiency than soil samples at the same site (Ryan et al. 2022). Artificial setups, such as water tanks in arid environments, can further enhance detectability of specific species as they attract wildlife to specific locations. Sampling eDNA from these sites can be a valuable complementary monitoring method to camera trapping or manual observation, and can lead to the detection of additional taxa (Kim et al. 2022). For instance, eDNA collection efforts from shelters designed for cryptic reptiles have significantly outperformed observation‐based surveys (Kyle et al. 2022; Matthias et al. 2021).

##### 4.3.2.1 Properties and Limitations

Sampling eDNA from species attraction sites, such as shelters and feeding sites, can lead to effective detection of target species with a comparable low search effort. Compared with visual detection methods, this does not require getting lucky with the timing to directly observe the species at the site, which is particularly useful for small, shy and nocturnal animals. On the downside, this might cause considerable uncertainty about the actual timing of the species' presence (temporal deviation), which can complicate precise biomonitoring. Some studies exist that have explored the extent of temporal deviation at specific types of attraction sites and found that for artificial water tanks, eDNA is only detectable for 3–12 h after a species' presence, due to rapid DNA degradation in water (Farrell et al. 2022). Sediments accumulated in bird nests, on the other hand, detect species for up to 2 weeks after the last visual observation (Kyle et al. 2022). Further studies are needed to fully explore the possible extent of temporal deviation, especially in the case of nests and shelters that may be occupied by multiple consecutive species, being used for long periods of time.

## 5 General Reflections

When planning an eDNA monitoring study, it is important to clearly define the target species or taxonomic group, as this influences both the types of substrates to sample and the timing of sample collection. If the primary goal is to monitor a specific species or group of species, researchers should tailor their approach around the ecological characteristics of those targets. Beyond the principles of eDNA ecology, it is essential to account for the life cycles and behaviours of target organisms, since recurring biological events, such as breeding, migration and hibernation, can strongly affect eDNA availability in different substrates. A solid understanding of the species' ecology, including their movement patterns and habitat preferences, enables more targeted sampling of substrates from environments they are most likely to occupy. By integrating ecological knowledge with methodological planning, researchers can make informed choices that enhance the effectiveness and accuracy of eDNA‐based monitoring.

Yet in other cases, the goal of eDNA biomonitoring projects can be general diversity inventories with broad taxonomic coverage, for example when assessing ecosystem health and evaluating the efficacy of conservation measures or the impacts of land‐use changes (Barnes and Turner 2015; Lock et al. 2022; Myers et al. 2000). In these cases where eDNA sampling is used for biodiversity inventories, it is most essential that the sampling substrates and techniques, as well as laboratory and bioinformatic workflows, are kept consistent between sampling sites and sampling intervals to ensure comparability between eDNA inventory results. Particularly when evaluating spatially or temporally explicit habitat modifications, such as land‐use changes or human modifications of existing habitats, it is essential to account for the potential of temporal and spatial deviation. For example, soil samples generally provide a spatially explicit insight into the local species community but may still contain traces of DNA from taxa that were only present before the impact and have since vanished. Air filters, on the other hand, are less prone to pick up DNA signals of the past but instead may not be as spatially explicit and pick up DNA evidence of species that exist only in nearby intact sites. These challenges can be mitigated to some extent. The temporal deviation in soil samples can be reduced by sequencing environmental RNA, which generally has a lower preservation potential than DNA and therefore more accurately detects evidence of currently active species. Similarly, the spatial deviation of air filters can be reduced by placing them lower to the ground, leading to a reduced probability of picking up DNA from other sites.

## 6 Future Perspectives

Terrestrial eDNA analyses hold great promise for transforming biodiversity monitoring, yet several critical challenges and unknowns remain, which must be addressed to fully realise this potential. Current limitations stem from our incomplete understanding of eDNA ecology in various substrates, from a lack of standardisation in eDNA sampling and processing methods, and from current technological limitations (Appendix S1) related to sequencing depth for detecting rare species against complex environmental backgrounds (Bohmann and Lynggaard 2023; Cowgill et al. 2024; Newton et al. 2025).

A fundamental priority lies in advancing our knowledge of eDNA ecology, particularly how DNA persists, degrades and moves through different substrates such as soil and air, as well as along biological substrates such as plants and invertebrates. The dispersal of eDNA in aquatic environments has been extensively studied, yet accurately predicting its original source or the transport route remains challenging, even when water currents are well characterised (Sakata et al. 2021). eDNA transport is potentially even more complex in terrestrial systems, necessitating further explorations (Bohmann and Lynggaard 2023; van der Heyde et al. 2020). Standardising methodological approaches for each substrate represents an equally pressing need. Current variability in sampling protocols, preservation techniques and molecular methods introduce substantial inconsistencies when comparing results across studies. Research efforts should focus on identifying optimal sampling strategies that account for ecosystem‐specific characteristics and target taxa. Developing ecosystem‐specific best practices, validated through rigorous interlaboratory comparisons, will be essential for building reliable monitoring networks and enabling robust meta‐analyses across temporal and spatial scales.

With increasing knowledge of eDNA ecology and standardisation of eDNA sampling campaigns, we will improve the interpretability and interoperability of eDNA datasets collected at different sites across the world. This will allow us to take full advantage of the scalability of eDNA monitoring, as eDNA sampling programs can leverage citizen science or other long‐term monitoring initiatives to increase the number of sites and frequency for biodiversity monitoring. The simplicity of many eDNA sampling approaches allows non‐experts to participate in sample collecting, contributing valuable data to scientific research. In addition, organisations with established field sites can integrate eDNA monitoring into their existing data collection schemes (Egeter et al. 2018; Palacios Mejia et al. 2021). By leveraging existing resources and engaging a large number of sampling agents (e.g., volunteers), eDNA‐based detection can enhance biodiversity assessments while reducing logistical costs (Corrales et al. 2025). Careful planning of such sampling efforts remains crucial, considering the substrate‐specific properties and challenges with eDNA biodiversity monitoring outlined in this review.

## Funding

This work was supported by the Swedish Research Council, 2023‐05366, and the SciLifeLab & Wallenberg Data Driven Life Science Program, KAW 2020.0239.

## Conflicts of Interest

The authors declare no conflicts of interest.

## Supporting information


**Table S1:** Detailed information of selected studies.

**Appendix S1:** eDNA sample preservation and processing.

## Data Availability

All data related to this review paper are available in Table 1, as well as the Appendix S1 and Table S1 (only available in the online version of this manuscript).
