# Supplementary material for: Properties and Limitations of eDNA Substrates for Terrestrial Animal Monitoring
Source: Mol Ecol Resour. 2026 Jan 23;26(2):e70096. doi: 10.1111/1755-0998.70096 (PMC12831013; doi:10.1111/1755-0998.70096)
Supplement: Supplementary file 2 — Appendix S1: eDNA sample preservation and processing. [file MEN-26-e70096-s001.pdf]

Appendix 1 – eDNA sample preservation and processing

Sample preservation

The preservation and initial processing of eDNA substrates are critical steps that directly influence detection accuracy and reliability (Cunningham et al., 2024). Substrate-specific handling is essential to minimize DNA degradation, contamination, and loss, all of which present unique challenges that can impact downstream results (Table 2). Proper protocols must account for environmental conditions, substrate composition, and logistical constraints to ensure data integrity.

For water samples, immediate filtration in the field is commonly applied to capture eDNA before degradation occurs (K. Bell et al., 2025; Gutiérrez-López et al., 2023). Filter pore size represents a key trade-off; smaller pores (e.g., 0.2-0.45 µm) retain more DNA but clog quickly in turbid water, reducing filtration efficiency (Turner et al., 2014; Zhao et al., 2021). After filtration, preservatives like hexadecyltrimethylammonium (CTAB) stabilize DNA by inhibiting nuclease activity, enabling room-temperature transport for short periods up to two weeks (Renshaw et al., 2015; Rieder et al., 2024). For long-term storage, freezing at −20°C or −80°C remains optimal, since delays in processing especially in warm or nutrient-rich water can lead to rapid microbial degradation (van Bochove et al., 2020; Zhao et al., 2023).

Solid abiotic substrates such as soil and sediment require specialized handling due to their heterogeneous composition. DNA binds tightly to soil particles, which protects it from degradation but can simultaneously reduce extraction efficiency (Cai et al., 2006). Freezing soil at −20°C is widely adopted, though field preservation via silica beads offers a practical alternative for remote sampling (Hermans et al., 2022; Tetzlaff et al., 2024). Inhibitors like humic acids are pervasive in clay-rich soils and necessitate additional purification steps, such as polyvinylpolypyrrolidone (PVPP) treatment (Arbeli & Fuentes, 2007) or specialized extraction kits (Cai et al., 2006).

Biotic substrates demand rapid stabilization to counteract enzymatic and microbial degradation. Most biotic materials (e.g., spiderwebs, plant tissues, scats) are field-preserved with cooling blocks and transferred to −20°C as soon as possible, typically within hours. Spiderwebs, for instance, require gentle handling to avoid fragmentation and should be swabbed with sterile tools to minimize contamination (Gregorič et al., 2022; Xu et al., 2015). Plant surfaces often harbor transient eDNA from pollinators or herbivores, necessitating immediate freezing to preserve trace DNA (M. D. Johnson, Katz, et al., 2023; Jønsson et al., 2023). Scats are particularly prone to bacterial degradation (Chiu-Werner & Jones, 2023; Thomassen et al., 2023). In some studies, invertebrates such as blood-feeding leeches or carrion flies were stored

in ethanol (70–95%) instead of cold preservation (Fernandes et al., 2023; Lynggaard et al., 2019; Saranholi et al., 2023).

Table A1. General preservation methods for different environmental DNA (eDNA) materials during transportation in the field.

Material	Preservation during transportation	Consideration
**Water**		
Field water, rain washes, artificial setup water	After on-site filtration, filter stored in CTAB buffer or similar at room-temperature for up to two weeks (Shuai et al., 2025)	Immediate filtration to prevent DNA degradation
**Solid abiotic substrates**		
Air	Passive or active air filter stored in sterile tubes at room temperature (Garrett, Watkins, Francis, et al., 2023)	Avoid contamination during handling
Sediment, soil, footprint	Cold preservation (Lilja et al., 2023), or at room-temperature (Marquina et al., 2019), or dry by silica beads (Dyson et al., 2024)	Extra DNA purification steps to remove inhibitors
Swab	Preserve cotton swab in sterile RNAlater solution (Lyman et al., 2022)	Ensure complete immersion of swab for effective preservation
**Biotic substrates**		
Plant tissue, animal tissue, spiderweb, scat	Cold preservation (Stothut et al., 2024)	Low temperature to avoid DNA degradation
Invertebrate	Stored in ethanol (70–95%), or at room-temperature (Saranholi et al., 2023)	Low temperature or ethanol to avoid DNA degradation

Impact of molecular methods

Multiple molecular detection methods can be applied in eDNA studies. The choice of method significantly influences the accuracy, sensitivity, and scope of eDNA-based monitoring, with each approach presenting distinct advantages and limitations.

Targeting specific species

Quantitative PCR (qPCR) and droplet digital PCR (ddPCR) are widely used for targeted species detection due to their exceptional sensitivity (detecting as few as 1–10 DNA copies/µL for qPCR and 0.1–1 copies/µL for ddPCR) and precise quantification capabilities (Doi et al., 2015). While both methods amplify short target sequences (typically 50–150 bp), they differ fundamentally: qPCR measures fluorescence accumulation during amplification cycles relative to a standard curve, while ddPCR partitions the sample into thousands of nanodroplets to count positive and negative reactions (Miotke et al., 2014). This partitioning allows ddPCR to perform better with low-abundance targets (<10 copies/µL) and samples containing PCR inhibitors, providing absolute quantification without the need for calibration standards (Doi et al., 2015; Takasaki et al., 2021).

These techniques are indispensable for applications demanding high specificity, such as monitoring endangered species or early-stage invasive species surveillance (Kirtane et al., 2024). Unlike metabarcoding, these methods deliver species-specific results without DNA sequencing, making them ideal for rapid decision-making in conservation or biosecurity. Additionally, these specific-targeting methods outperform metabarcoding in detection rate, as shown by Allen et al. in a study monitoring bats (Allen et al., 2023). However, their reliance on species-specific primers limits scalability for community-level studies. Therefore, rigorous validation is essential, primers should be tested *in silico* (e.g., using NCBI Primer-BLAST) and *in vitro* with positive and negative controls to minimize false-negatives from primer mismatches (Nishizawa et al., 2023; Thalinger et al., 2021). False positives can be minimized by melt curve analysis (qPCR) or threshold optimization (ddPCR), alongside the use of blank controls and technical replicates (Mauvisseau et al., 2019).

Conventional PCR with Sanger sequencing remains relevant despite lower quantification sensitivity, primarily because it can amplify longer fragments (up to 1000 bp) compared to less than 200 bp in qPCR and ddPCR. It is particularly advantageous for verifying questionable qPCRor ddPCR results, through direct sequencing, analyzing genomic regions that require longer amplicons, and offering a cost-effective solution for small-scale projects where high-throughput methods are impractical (Dittrich et al., 2023; Hebert et al., 2018; Monge et al., 2020). Although less sensitive, it offers higher sequence certainty and reduced primer bias, making it indispensable when accuracy outweighs rapid quantification. Its continued utility in targeted research complements rather than competes with newer technologies, each serving distinct but equally important roles in molecular detection (Quasim et al., 2018).

Targeting biodiversity

Metabarcoding has revolutionized biodiversity assessment by enabling high-throughput detection of multiple taxa through standardized genetic markers (Cowgill et al., 2024; Rey et al., 2019), though it faces inherent limitations in species coverage and quantitative accuracy due to primer-template interactions. Commonly used universal primers for specific barcode regions (e.g., COI, 16S, or ITS regions) typically capture 70-90% of expected taxa within a target group but often miss divergent lineages or species with primer-binding site mutations (Takenaka et al., 2024; Zhang & Bu, 2022). The method's quantitative reliability is further compromised by taxon-specific amplification efficiencies, differences in gene copy numbers, primer binding affinity, and PCR competition, can distort relative abundance estimates, sometimes by 10-100-fold (Elbrecht et al., 2018; Nichols et al., 2018). These constraints also hinder the detection of rare species in eDNA samples, as dominant taxa can monopolize sequencing resources (Takasaki et al., 2021). Nevertheless, when applied with awareness of these limitations, metabarcoding remains unmatched for revealing community structure and discovering unexpected taxa, making it

indispensable for exploratory biodiversity studies where predefined targets are impractical (Berry et al., 2017; Ritter et al., 2018).

Metagenomics or shotgun metagenomics, which sequences all DNA molecules in a sample, provides the most comprehensive biodiversity assessment without target-specific amplification (Curto et al., 2025; Seeber & Epp, 2022). Unlike metabarcoding, it avoids primer biases and captures both taxonomic and functional genetic information (Mirete et al., 2016; Serite et al., 2023). However, because microbial DNA often constitutes over 90% of eDNA in most samples, detecting rare macroscopic species requires intensive sequencing efforts (Khan et al., 2025). This combined with high costs and computational demands, makes metagenomics impractical for large-scale monitoring. Its primary applications remain microbial community analysis, functional gene discovery, and situations where comprehensive genetic information justifies the cost (Willms et al., 2021).

Recent innovations related to DNA sequencing techniques are crucial to address current limitations in eDNA-based monitoring of terrestrial animals, particularly in terms of sensitivity and taxonomic resolution (Magoga et al., 2022; Nichols et al., 2018). Emerging approaches that combine CRISPR-based target enrichment with high-throughput sequencing hold promise for overcoming key challenges in metabarcoding and metagenomics. These challenges include PCR bias and the low recovery of animal DNA from microbial-dominated samples (Malekshoar et al., 2023; Phelps, 2019; M. Williams et al., 2019; M. A. Williams et al., 2022). Instead of PCR amplification, CRISPR-based Cas9-sgRNA enables sequence-specific capture of low-abundance DNA, thereby enhancing the detection of elusive or rare terrestrial species (McDonald et al., 2021). Furthermore, the development of portable, field-deployable real-time sequencing systems such as Nanopore MinION has the potential to facilitate on-site biodiversity assessments (Wang et al., 2021).

References

Bell, K., Evans, M. J., Lindenmayer, D. B., Scheele, B. C., Smith, D. G., & Malerba, M. E. (2025). Excluding livestock from farm dams enhances native biodiversity. *Agriculture, Ecosystems & Environment*, *386*, 109623. https://doi.org/10.1016/J.AGEE.2025.109623

Berry, T. E., Osterrieder, S. K., Murray, D. C., Coghlan, M. L., Richardson, A. J., Grealy, A. K., Stat, M., Bejder, L., & Bunce, M. (2017). DNA metabarcoding for diet analysis and biodiversity: A case study using the endangered Australian sea lion (Neophoca cinerea). *Ecology and Evolution*, *7*(14), 5435–5453. https://doi.org/10.1002/ECE3.3123

Chiu-Werner, A., & Jones, M. (2023). Human land-use changes the diets of sympatric native and invasive mammal species. *Ecology and Evolution*, *13*(12), e10800. https://doi.org/10.1002/ECE3.10800

Cunningham, S. W., Tessler, M., Johnson-Rosemond, J., Whittaker, I. S., & Brugler, M. R. (2024). Environmental DNA Isolation, Validation, and Preservation Methods. *Methods in Molecular Biology*, *2744*, 171–180. https://doi.org/10.1007/978-1-0716-3581-0_10

Curto, M., Veríssimo, A., Riccioni, G., Santos, C. D., Ribeiro, F., Jentoft, S., Alves, M. J., & Gante, H. F. (2025). Improving Whole Biodiversity Monitoring and Discovery With Environmental DNA Metagenomics. *Molecular Ecology Resources*, e14105. https://doi.org/https://doi.org/10.1111/1755-0998.14105

Dittrich, A., Lang, J., Schütz, C., Sittler, B., & Eitzinger, B. (2023). Identifying invertebrate species in Arctic muskox dung using DNA barcoding. *Polar Research*, *42*. https://doi.org/10.33265/polar.v42.9017

Doi, H., Takahara, T., Minamoto, T., Matsuhashi, S., Uchii, K., & Yamanaka, H. (2015). Droplet digital polymerase chain reaction (PCR) outperforms real-time PCR in the detection of environmental DNA from an invasive fish species. *Environmental Science and Technology*, *49*(9), 5601–5608. https://doi.org/10.1021/acs.est.5b00253

Dyson, K., Nicolau, A. P., Tenneson, K., Francesconi, W., Daniels, A., Andrich, G., Caldas, B., Castaño, S., de Campos, N., Dilger, J., Guidotti, V., Jaques, I., McCullough, I. M., McDevitt, A. D., Molina, L., Nekorchuk, D. M., Newberry, T., Pereira, C. L., Perez, J., … Saah, D. (2024). Coupling remote sensing and eDNA to monitor environmental impact: A pilot to quantify the environmental benefits of sustainable agriculture in the Brazilian Amazon. *PLOS ONE*, *19*(2), e0289437. https://doi.org/10.1371/JOURNAL.PONE.0289437

Elbrecht, V., Vamos, E. E., Steinke, D., & Leese, F. (2018). Estimating intraspecific genetic diversity from community DNA metabarcoding data. *PeerJ*, *2018*(4), e4644. https://doi.org/10.7717/PEERJ.4644/SUPP-8

Garrett, N. R., Watkins, J., Francis, C. M., Simmons, N. B., Ivanova, N., Naaum, A., Briscoe, A., Drinkwater, R., & Clare, E. L. (2023). Out of thin air: surveying tropical bat roosts through air sampling of eDNA. *PeerJ*, *11*. https://doi.org/10.7717/peerj.14772

Hebert, P. D. N., Braukmann, T. W. A., Prosser, S. W. J., Ratnasingham, S., deWaard, J. R., Ivanova, N. V., Janzen, D. H., Hallwachs, W., Naik, S., Sones, J. E., & Zakharov, E. V. (2018). A Sequel to Sanger: Amplicon sequencing that scales. *BMC Genomics*, *19*(1), 1–14. https://doi.org/10.1186/S12864-018-4611-3/FIGURES/10

Hermans, S. M., Lear, G., Buckley, T. R., & Buckley, H. L. (2022). Environmental DNA sampling detects between-habitat variation in soil arthropod communities, but is a poor indicator of fine-scale spatial and seasonal variation. *Ecological Indicators*, *140*. https://doi.org/10.1016/j.ecolind.2022.109040

Khan, A., Carter, R., Mpamhanga, C. D., Masiga, D., Channumsin, M., Ciosi, M., Manangwa, O., Mramba, F., Ijaz, U. Z., Auty, H., & Mable, B. K. (2025). Swatting Flies: Biting Insects as Non-Invasive Samplers for Mammalian Population Genomics. *Molecular Ecology*, *n/a*(n/a), e17661. https://doi.org/https://doi.org/10.1111/mec.17661

Kirtane, A., Howard, L., Beaver, C. E., Hunter, M. E., Luikart, G., & Deiner, K. (2024). How, What, and Where You Sample Environmental DNA Affects Diversity Estimates and Species Detection. *Environmental DNA*, *6*(6). https://doi.org/10.1002/edn3.70042

Lilja, M. A., Buivydaitė, Ž., Zervas, A., Krogh, P. H., Hansen, B. W., Winding, A., & Sapkota, R. (2023). Comparing earthworm biodiversity estimated by DNA metabarcoding and morphology-based approaches. *Applied Soil Ecology*, *185*, 104798. https://doi.org/10.1016/J.APSOIL.2022.104798

Magoga, G., Forni, G., Brunetti, M., Meral, A., Spada, A., De Biase, A., & Montagna, M. (2022). Curation of a reference database of COI sequences for insect identification through DNA metabarcoding: COins. *Database*, *2022*, 55. https://doi.org/10.1093/DATABASE/BAAC055

Malekshoar, M., Azimi, S. A., Kaki, A., Mousazadeh, L., Motaei, J., & Vatankhah, M. (2023). CRISPR-Cas9 Targeted Enrichment and Next-Generation Sequencing for Mutation Detection. *The Journal of Molecular Diagnostics*, *25*(5), 249–262. https://doi.org/10.1016/J.JMOLDX.2023.01.010

Mauvisseau, Q., Davy-Bowker, J., Bulling, M., Brys, R., Neyrinck, S., Troth, C., & Sweet, M. (2019). Combining ddPCR and environmental DNA to improve detection capabilities of a critically endangered freshwater invertebrate. *Scientific Reports*, *9*(1), 1–9. https://doi.org/10.1038/s41598-019-50571-9

McDonald, T. L., Zhou, W., Castro, C. P., Mumm, C., Switzenberg, J. A., Mills, R. E., & Boyle, A. P. (2021). Cas9 targeted enrichment of mobile elements using nanopore sequencing. *Nature Communications 2021 12:1*, *12*(1), 1–13. https://doi.org/10.1038/s41467-021-23918-y

Miotke, L., Lau, B. T., Rumma, R. T., & Ji, H. P. (2014). High sensitivity detection and quantitation of DNA copy number and single nucleotide variants with single color droplet digital PCR. *Analytical Chemistry*, *86*(5), 2618–2624. https://doi.org/10.1021/ac403843j

Mirete, S., Morgante, V., & González-Pastor, J. E. (2016). Functional metagenomics of extreme environments. In *Current Opinion in Biotechnology* (Vol. 38, pp. 143–149). Elsevier Ltd. https://doi.org/10.1016/j.copbio.2016.01.017

Nichols, R. V., Vollmers, C., Newsom, L. A., Wang, Y., Heintzman, P. D., Leighton, M., Green, R. E., & Shapiro, B. (2018). Minimizing polymerase biases in metabarcoding. *Molecular Ecology Resources*, *18*(5), 927–939. https://doi.org/10.1111/1755-0998.12895

Nishizawa, R., Nakao, R., Ushimaru, A., & Minamoto, T. (2023). Development of environmental DNA detection assays for snakes in paddy fields in Japan. *Landscape and Ecological Engineering*, *19*(1), 3–10. https://doi.org/10.1007/s11355-022-00496-9

Phelps, M. (2019). Increasing eDNA capabilities with CRISPR technology for real-time monitoring of ecosystem biodiversity. *Molecular Ecology Resources*, *19*(5), 1103–1105. https://doi.org/10.1111/1755-0998.13084

Renshaw, M. A., Olds, B. P., Jerde, C. L., Mcveigh, M. M., & Lodge, D. M. (2015). The room temperature preservation of filtered environmental DNA samples and assimilation into a phenol–chloroform–isoamyl alcohol DNA extraction. *Molecular Ecology Resources*, *15*(1), 168–176. https://doi.org/10.1111/1755-0998.12281

Rey, A., Carney, K. J., Quinones, L. E., Pagenkopp Lohan, K. M., Ruiz, G. M., Basurko, O. C., & Rodríguez-Ezpeleta, N. (2019). Environmental DNA Metabarcoding: A Promising Tool for Ballast Water Monitoring. *Environmental Science and Technology*, *53*(20), 11849–11859. https://doi.org/10.1021/acs.est.9b01855

Rieder, J., Jemmi, E., Hunter, M. E., & Adrian-Kalchhauser, I. (2024). A Guide to Environmental DNA Extractions for Non-Molecular Trained Biologists, Ecologists, and Conservation Scientists. *Environmental DNA*, *6*(5), e70002. https://doi.org/10.1002/EDN3.70002

Seeber, P. A., & Epp, L. S. (2022). Environmental DNA and metagenomics of terrestrial mammals as keystone taxa of recent and past ecosystems. *Mammal Review*, *52*(4), 538–553. https://doi.org/https://doi.org/10.1111/mam.12302

Serite, C. P., Emami-Khoyi, A., Ntshudisane, O. K., James, N. C., Jansen van Vuuren, B., Bodill, T., Cowley, P. D., Whitfield, A. K., & Teske, P. R. (2023). eDNA metabarcoding vs metagenomics: an assessment of dietary competition in two estuarine pipefishes. *Frontiers in Marine Science*, *Volume 10-2023*. https://www.frontiersin.org/journals/marine-science/articles/10.3389/fmars.2023.1116741

Shuai, H., Liu, X., Zhang, Y., Sun, Y., Li, H., & Li, Z. (2025). eDNA enhances detection efficiency but reveals lower waterbird diversity: A comparison with point counting method. *Avian Research*, *16*(2), 100236. https://doi.org/10.1016/J.AVRS.2025.100236

Stothut, M., Kühne, D., Ströbele, V., Mahla, L., Künzel, S., & Krehenwinkel, H. (2024). Environmental DNA metabarcoding reliably recovers arthropod interactions which are frequently observed by video recordings of flowers. *Environmental DNA*, *6*(3), e550. https://doi.org/10.1002/EDN3.550

Takenaka, M., Hasebe, Y., Yano, K., Okamoto, S., Tojo, K., Seki, M., Sekiguchi, S., Jitsumasa, T., Morohashi, N., Handa, Y., & Sakaba, T. (2024). Environmental DNA metabarcoding on aquatic

insects: Comparing the primer sets of MtInsects-16S based on the mtDNA 16S and general marker based on the mtDNA COI region. *Environmental DNA*, *6*(4), e588. https://doi.org/10.1002/EDN3.588

Thalinger, B., Deiner, K., Harper, L. R., Rees, H. C., Blackman, R. C., Sint, D., Traugott, M., Goldberg, C. S., & Bruce, K. (2021). A validation scale to determine the readiness of environmental DNA assays for routine species monitoring. *Environmental DNA*, *3*(4), 823–836. https://doi.org/10.1002/EDN3.189

Thomassen, E. E., Sigsgaard, E. E., Jensen, M. R., Olsen, K., Hansen, M. D. D., Svenning, J. C., & Thomsen, P. F. (2023). Contrasting seasonal patterns in diet and dung-associated invertebrates of feral cattle and horses in a rewilding area. *Molecular Ecology*, *32*(8), 2071–2091. https://doi.org/10.1111/MEC.16847

Turner, C. R., Barnes, M. A., Xu, C. C. Y., Jones, S. E., Jerde, C. L., & Lodge, D. M. (2014). Particle size distribution and optimal capture of aqueous macrobial eDNA. *Methods in Ecology and Evolution*, *5*(7), 676–684. https://doi.org/10.1111/2041-210X.12206

van Bochove, K., Bakker, F. T., Beentjes, K. K., Hemerik, L., Vos, R. A., & Gravendeel, B. (2020). Organic matter reduces the amount of detectable environmental DNA in freshwater. *Ecology and Evolution*, *10*(8), 3647–3654. https://doi.org/10.1002/ECE3.6123

Williams, M. A., Eyto, E. de, Caestecker, S., Regan, F., & Parle-McDermott, A. (2022). Development and field validation of RPA-CRISPR-Cas environmental DNA assays for the detection of brown trout (Salmo trutta) and Arctic char (Salvelinus alpinus). *Environmental DNA*. https://doi.org/10.1002/EDN3.384

Williams, M., O'Grady, J., Ball, B., Carlsson, J., Eyto, E., McGinnity, P., Jennings, E., Regan, F., & Parle-McDermott, A. (2019). The application of CRISPR-Cas for single species identification from environmental DNA. *Molecular Ecology Resources*, *19*(5), 1106–1114. https://doi.org/10.1111/1755-0998.13045

Willms, I. M., Grote, M., Kocatürk, M., Singhoff, L., Kraft, A. A., Bolz, S. H., & Nacke, H. (2021). Novel soil-derived beta-lactam, chloramphenicol, fosfomycin and trimethoprim resistance genes revealed by functional metagenomics. *Antibiotics*, *10*(4). https://doi.org/10.3390/ANTIBIOTICS10040378/S1

Zhang, H., & Bu, W. (2022). Exploring Large-Scale Patterns of Genetic Variation in the COI Gene among Insecta: Implications for DNA Barcoding and Threshold-Based Species Delimitation Studies. *Insects*, *13*(5), 425. https://doi.org/10.3390/INSECTS13050425/S1
